# Effects of lipopolysaccharide-induced septic shock on rat isolated kidney, possible role of nitric oxide and protein kinase C pathways

**DOI:** 10.22038/IJBMS.2018.27798.6773

**Published:** 2018-10

**Authors:** Zahra Gholamnezhad, Zahra Fatehi Hassanabad

**Affiliations:** 1 Neurogenic Inflammation Research Center, Mashhad University of Medical Sciences, Mashhad, Iran; 2 Department of Physiology, Faculty of Medicine, Mashhad University of Medical Sciences, Mashhad, Iran; 3 Emam zaman Hospital, Mashhad, Iran

**Keywords:** Kidney, Lipopolysaccharide, Nitric oxide, Protein kinase C, Rat, Vasoconstrictor, Vasodilator

## Abstract

**Objective(s):**

Pathophysiology of sepsis-associated renal failure (one of the most common cause of death in intensive care units) had not been fully determined. The effect of nitric oxide and protein kinase C (PKC) pathways in isolated kidney of Lipopolysaccharide-treated (LPS) rats were investigated in this study.

**Materials and Methods:**

Vascular responsiveness to phenylephrine and acetylcholine in the presence and absence of a potent PKC inhibitor (chelerythrine) and nonspecific NO inhibitor (L-NAME) as well as responses to acetylcholine and sodium nitroprusside (SNP) were examined.

**Results:**

LPS (10 mg/kg, IP) treatment resulted in a lower systemic pressure and reduction of responses to vasoconstrictor and vasodilator agents (*P*<0.05 to *P*<0.01). The contractile response to phenylephrine and the relaxation response to acetylcholine were significantly blunted in isolated kidneys removed from LPS-treated rats. L-NAME (10 µM) preincubation modified the responses to acetylcholine in isolated kidneys of control animal (*P*<0.001) but not in LPS-treated rats. While, chelerythrine (10 µM) preincubation partially restored response to phenylephrine in LPS-treated tissues.

**Conclusion:**

Present study highlighted that five hours of intraperitoneal endotoxin injection is adequate to reduce renal basal perfusion pressure. These results also suggest that PKC inhibition may have a beneficial role in vascular hyporesponsiveness induced by LPS. Although our study partly elaborated on the effects of LPS on isolated renal vascular responses to vasoactive agents, further studies are required to explain how LPS exerts its renal vascular effects.

## Introduction

Sepsis is a systemic inflammatory response to invading microorganism and microbial toxins, such as lipopolysaccharide (LPS) endotoxin of Gram-negative bacteria. LPS causes the release of potent proinflammatory mediators, which in turn are largely responsible for cardiovascular derangement, multiple organ failure, shock, and death (1, 2). In the United States, an estimated 700,000 cases of sepsis occur each year, resulting in more than 200,000 deaths, which accounts for 10 percent of all deaths annually and exceeds the number of deaths due to myocardial infarction (3).

Despite the fact that sepsis is characterized by a decrease in systemic vascular resistance, the main cause of death is due to multiple organ failure (4). Furthermore, acute renal failure (ARF) occurs in approximately 50% of patients with septic shock which is associated with a high mortality rate despite different therapies including dialysis (5). Moreover, it has been speculated that renal failure has a significant role in inducing organ damage in sepsis (4). Sepsis caused by Gram-negative bacteria leads to hemodynamic changes, including hypotension; reduction in renal blood flow and also global and regional ischemia which may lead to ARF (6, 7). In addition, renal perfusion pressure reduces which is thought to be partly due to a preglomerular vasoconstriction, it consequently plays a major role in the development of sepsis-associated renal failure (8).

While it appears that endotoxin triggers a series of humoral and cellular reactions which result in the unregulated production of secondary mediators capable of initiating renal hypoperfusion, the exact pathogenesis of renal microvascular response is not fully understood (8). One of the most important mediators in this phenomenon is overproduction of nitric oxide (NO) caused by increased expression of the inducible form of nitric oxide synthase (9). This increase occurs in many types of cells, including vascular smooth-muscle cells and endothelial cells (10-12). 

Previously, we have shown that protein kinase C (PKC) and NO pathways are altered in aortic rings of LPS-treated rats and chelerythrine (a potent PKC inhibitor) pretreatment was able to significantly improve the LPS-induced endothelial dysfunction (13). In the current study, we have investigated whether LPS is able to modify renal vascular responses to vasodilators and vasoconstrictors; possible involvement of NO and PKC pathways are also studied.

## Materials and Methods

***Animals***

Adult male Sprague Dawley rats, (Razi Institutes, Mashhad, Iran) weighing between 250-300 g were used. Animals were housed in temperature and humidity controlled, light-cycled quarters. The protocols used conformed to guidelines for the conduct approved by the Committee on the Ethics of Animal Experiments in Mashhad University. A total of 40 rats were randomly divided into two main groups. Control rats received saline injection (1 ml/kg IP, n=20), whereas LPS-treated rats received a bolus injection of LPS (10 mg/kg IP, n=20) for five hours before examination. In each main group 5 rats had been examined for *in vivo* experiments and the remaining 15 rats were used for i*n vitro* experiments. 

***Experimental protocol***

*Drugs*

The following drugs were used; LPS, chelerythrine hydrochloride, acetylcholine chloride, heparin sodium, phenylephrine hydrochloride, NG-nitro-L-arginine methyl ester hydrochloride (L-NAME) and dimethyl sulphoxide (DMSO) were obtained from Sigma Laboratories. Sodium chloride, potassium chloride, magnesium sulphate, sodium hydrogen carbonate, potassium dihydrogen orthophosphate, D-glucose and calcium chloride were obtained from Merck Laboratories. Sodium thiopental was obtained from Biochemie GmbH Vienna, Austria. All solutions (except chelerythrine) were dissolved in distilled water and then diluted with normal saline. Chelerythrine was dissolved in DMSO (final dose or concentration of DMSO in the rats’ body and bathing solution was 0.01 ml/kg and 0.01%, respectively). 

*Measurement of arterial blood pressure*

After five hours of LPS or saline injection, arterial blood pressure was measured as previously described (14). Briefly, rats were anaesthetized with sodium thiopental (45 mg/kg by IP injection) and the right common carotid artery was catheterized for the measurement of blood pressure. Left jugular vein was also catheterized for administration of additional sodium thiopental, and IV injection of *in vivo* experimental drug, including phenylephrine, acetylcholine, and sodium nitroprusside (SNP). The trachea was also cannulated to facilitate spontaneous respiration. Animals were allowed to stabilize for 20 min prior to data collection. Body temperature was recorded using a rectal thermistor probe and was maintained at 37 ± 0.5 °C using an incandescent lamp placed over the abdomen. After stabilization period, arterial blood pressure (systolic, diastolic and mean) and heart rate were measured. In addition, *in vivo* pressor responses to phenylephrine (0.1 and 0.3 µg/kg) and depressor responses to acetylcholine (5 and 50 µg/kg), and SNP (0.01 and 0.1 µg/kg) had been evaluated.

*Studies on the isolated perfused kidney*

A full-length midline abdominal incision was made. In order to improve access to the renal vessels, the small and large intestine were pulled to the outside of the abdomen, superior mesenteric artery and vein, portal vein, third part of the duodenum and sigmoid colon with adjoining marginal vessels were individually looped with silk ligatures. The ligatures were tied; the mesenteric circulatory bed is isolated and the bowel and mesentery beyond the ligature were excised. Silk ligatures were placed around the aorta, two infrarenal and one suprarenal. The left renal vascular pedicles were gently dissected and the two ligatures were placed around the renal vein as it leaves the vena cava. Abdominal aorta in the distal infrarenal region was located and a small incision was made. Through this incision the perfusion cannula was inserted into the left renal artery and tied using the ligature. A short length of tubing (180 PE) was inserted into the renal vein and tied into place with the distal renal vein ligature. The kidney was removed and covered with wet gauzes to prevent the evaporative heat loss. The isolated kidney was placed in a petri-dish seated on the top of an organ bath (37 ^°^C); and the renal cannula was connected to the pressure transducer. The isolated kidney was perfused through this cannula using Krebs solution (mM): NaCl 118.4, KCl 4.7, MgSO_4_, H_2_O 1.2, KH_2_PO_4_, 2H_2_O, 1.2, NaHCO_3_ 25, CaCl_2_ 2.5, and glucose 11.1 in distilled water. This solution was maintained at 37 ^°^C, bubbled with 5% CO_2_ and 95% O_2_. Kidneys were perfused at a constant rate (7 ml/min; Gilson Minipuls 2). The perfusate, which flowed from the renal vein, was removed at a rate of 7 ml/min to prevent accumulation in the bath. The tissue was allowed to equilibrate for 30 min before commencing the experiments. Changes in arterial smooth muscle tension were recorded using a pressure transducer connected to a Wasinghton recorder (400 MD/2). 

The cumulative response curves for phenylephrine (10^-7^-10^-4^ M) were obtained in kidney isolated from control or LPS-treated animals. To evaluate the responses of isolated kidney to vasodilator agents, the preparations were contracted with phenylephrine (10^-5^ M), and then different concentrations of the acetylcholine (10^-7^- 10^-4^ M), or SNP (10^-9^-10^-6^ M) were added. To determine whether the effects of LPS are through the activation of the L-arginine- nitric oxide pathway or protein kinase C activation, some kidneys were incubated with either chelerythrine (10 µM) or L-NAME (10 µM) for 20 min prior to the addition of phenylephrine. 

*Data analysis and statistics*

Values in the text are presented as mean±SEM. Changes in mean arterial blood pressure were compared using Student’s t test. Differences between groups were compared using One Way ANOVA followed by a Tukey-Kramer multiple comparison test. A *P* value less than 0.05 was considered statistically significant.

## Results

***Hemodynamic effects of LPS (Escherichia coli endotoxin)***

The detailed results are given in Table 1. LPS (10 mg/kg) lowered systolic, diastolic and mean systemic arterial blood pressure; this decrease in pressure was significant after 5 hr of IP administration (mean arterial blood pressure; control: 118.78 ± 8.02 mmHg, LPS-treated: 70.65±3.07 mmHg, *P*<0.001 vs control). Heart rates were also significantly reduced by administration of LPS. 

***Effects of LPS treatment on responses to different agents in anaesthetized rats***

**Figure 1:**
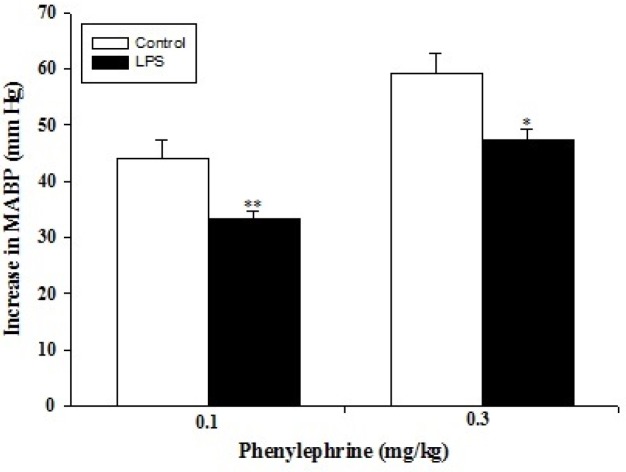
Increase in mean arterial blood pressure in response to phenylephrine (0.1 and 0.3 µg/kg) in control (n=5) and LPS-treated rats (n= 5). Data are presented as mean±SEM. * *P*<0.05; ** *P*<0.01 vs. control

**Figure 2:**
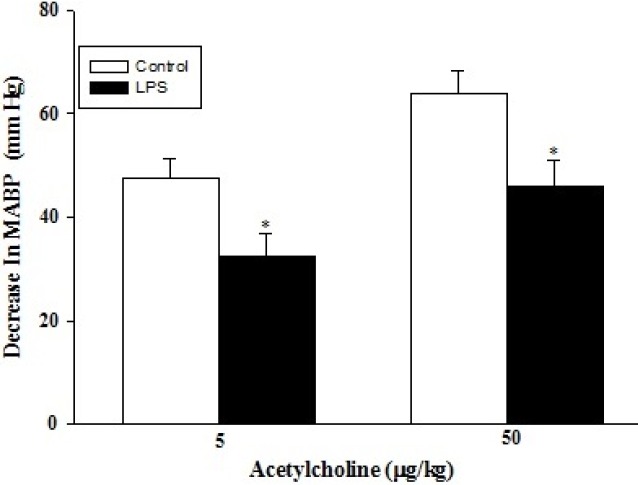
Decrease in mean arterial blood pressure in response to acetylcholine (5 and 50 µg/kg) in control (n=5) and LPS-treated rats (n= 5). Data are presented as mean±SEM. * *P*<0.05 vs. control

**Figure 3:**
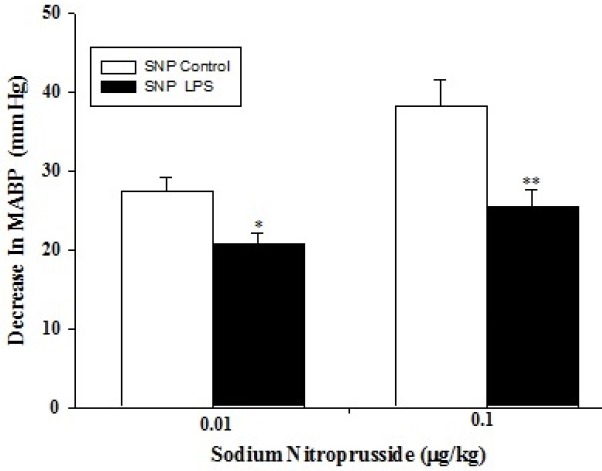
Decrease in mean arterial blood pressure in response to sodium nitroprusside (SNP; 0.01 and 0.1 µg/kg) in control (n=5) and LPS-treated rats (n=5). Data are presented as mean±SEM. * *P*<0.05; ** *P*<0.01 vs. control

Administration of phenylephrine (0.1 and 0.3 μg/kg) to the anaesthetized rats (either control, n=5 or LPS-treated, n=5) resulted in a dose-dependent increase in arterial blood pressure (Figure 1); which was more pronounced in the control group (*P*<0.05 and *P*<0.001 respectively). Intravenous bolus injection of acetylcholine (5 and 50 μg/kg) decreased arterial blood pressure in a dose-dependent manner (Figure 2), which were significantly lower in LPS-treated rats than those of control (*P*<0.05 for both doses). In addition, intravenous administration of SNP (0.01 and 0.1 μg/kg) resulted in a dose-dependent reduction in arterial blood pressure. LPS-treated rats showed a smaller vasodilatory response (*P*<0.05 and *P*<0.001 respectively) compared to those of control (Figure 3). 

***Effects of LPS treatment on responses to vasodilators and vasoconstrictors in rat isolated kidneys***

**Figure 4:**
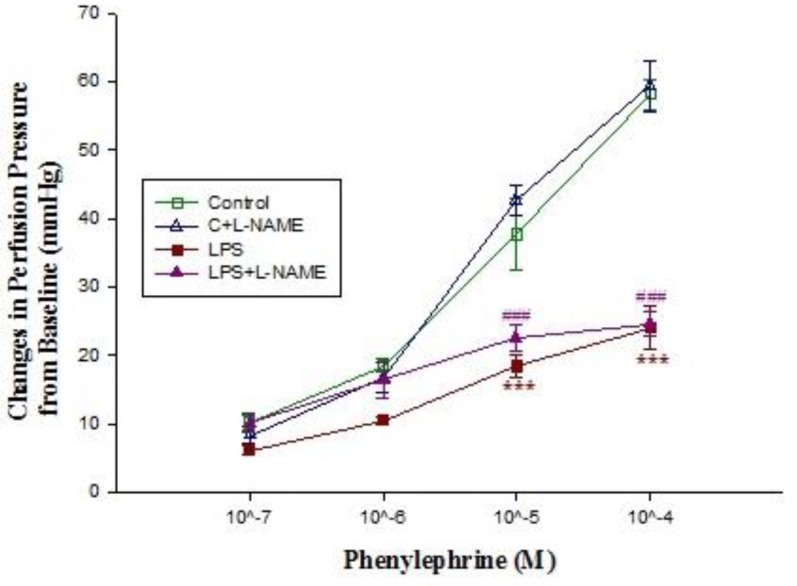
The concentration-response curves of phenylephrine-induced (10^-7^-10^-4^ M) contraction in isolated kidney from control (C; n=10) and LPS-treated rats (n= 10) in absence (n=5) and presence (n=5) of L-NAME incubation (10 μM for 20 min). Data are presented as mean±SEM. *** *P*<0.001 vs. control, ###; *P*<0.001 vs. control+L-NAME

Basal perfusion pressure in kidneys isolated from control rats was significantly higher than those of LPS-treated (*P*<0.05) (Table 1). Moreover, administration of different concentrations of phenylephrine (10^-5^-10^-4^ M) to Krebs’ solution markedly increased the perfusion pressure in kidneys removed from control rats but LPS pretreatment blunted the phenylephrine response (Figure 4). In phenylephrine pre-contracted kidneys, administration of acetylcholine (10^-6^ M) reduced the pressure**,** which was more pronounced in those that were removed from control rats (Figure 6). However, administration of SNP to the pre-contracted isolated kidneys caused a reduction in perfusion pressure which was almost similar among different groups (Figure 8). Pre-incubation of isolated kidneys with L-NAME (10 µM for 20 min) caused a non-significant change in basal perfusion pressure in tissues removed either from control rats (57.5±9 mmHg vs 70.6±5.1), or LPS-treated animals (46.7±1.7mmHg vs 47.2±1.4 mmHg; n.s). Responses to phenylephrine were not modified by L-NAME pre-incubation (Figure 4). However, incubation of phenylephrine pre-contracted kidneys with L-NAME modified the responses to acetylcholine (10^-6^-10^-5^ M) in isolated kidney’s of control animal (*P*<0.001) but not to those of LPS-treated rats (Figure 6). 

**Figure 5:**
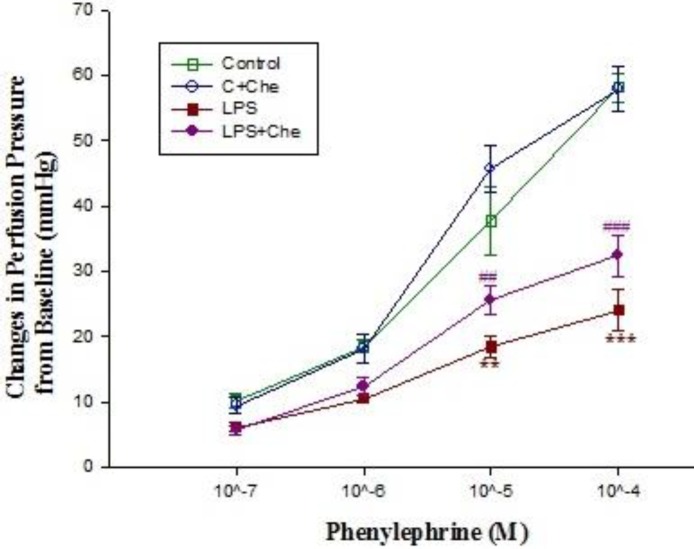
The concentration-response curves of phenylephrine-induced (10-7-10-4 M) contraction in isolated kidney from control (C; n=10) and LPS-treated rats (n= 10) in absence (n=5) and presence (n=5) of chelerythine (Che) incubation (10 μM for 20 min). Data are presented as mean±SEM. *** *P*<0.001 vs. control, ###; *P*<0.001 vs. control+ chelerythine

Addition of chelerythrine (10^-5^ M for 20 min) to the Krebs’ solution slightly increased the basal perfusion pressure to 52±1.4 mmHg from 47.2±1.4 mmHg in isolated tissues from LPS-treated rats but did not modify those of control (Tasble 1). Although chelerythrine did not modify the renal responses to phenylephrine in kidneys’ isolated from control rats**,** but it did partially restore phenylephrine responses in LPS-treated tissues (Figure 5). Responses to acetylcholine in phenylephrine pre-contracted tissues were moderately reduced by LPS pretreatment; chelerythrine (10^-5^ M) significantly augmented responses to 10^-5^ M of acetylcholine in kidneys removed from LPS-treated rats (Figure 7). In addition, SNP induced a concentration dependent relaxation in the phenylephrine precontracted isolated kidney which was almost similar in both groups (Figure 8).

## Discussion

The present study showed that intraperitoneal administration of LPS (*E. coli* endotoxin) to anesthetized rats not only induced a severe hypotension but it also decreased the systemic responses to phenylephrine. These effects of LPS have been extensively investigated and different mechanisms such as the reduction in the number of α-adrenoreceptors (15), over- production of different inflammatory mediators such as inducible nitric oxide (9), TNF- α (7) and cytokines have been shown (16) to be involved. Moreover, there is a relationship between proinflammatory mediator levels and hemodynamic responses to vasopressors (17). Considering the important role of renal functions during septic shock, we designed our experiments in a way to observe whether intraperitoneal injection of LPS can modify the renal vascular resistance as well as to investigate the renal responses to different vasoactive agents. As it was shown in the results section, basal perfusion pressure (BPP) of isolated kidneys removed from LPS-treated rats were significantly lower than control animals showing an increased renal vascular resistance. Pre-incubation of tissues with L-NAME or chelerythrine did not modify BPP**,** which may imply that NO overproduction and/or activation of PKC pathway are not the predominant factors responsible for renal hypoperfusion in LPS-treated rats. Even though our current study was not aimed to directly measure the systemic and renal vascular resistance in septic rats, we speculate that LPS pretreatment augmented the renal vascular resistance. This claim has been previously confirmed by Bougle and Duranteau 2011, who showed that systemic hypotension and decrease in total peripheral resistance as well as preglomerular vasoconstriction may decrease renal blood flow and perfusion pressure in septic condition (8).

**Table 1:** Basal perfusion pressure in the kidneys isolated from LPS-treated and control rats.

Groups	Control	Control+L-NAME	Control+Che	LPS-treated	LPS+L-NAME	LPS+Che
Basal perfusion pressure (mmHg)	70.6±5.1	57.5±9	72.2±9.6	47.2±1.4*	46.71.7	521.4

±Che: chelerythine.

* *P*< 0.05 *vs. *control.

**Figure 6:**
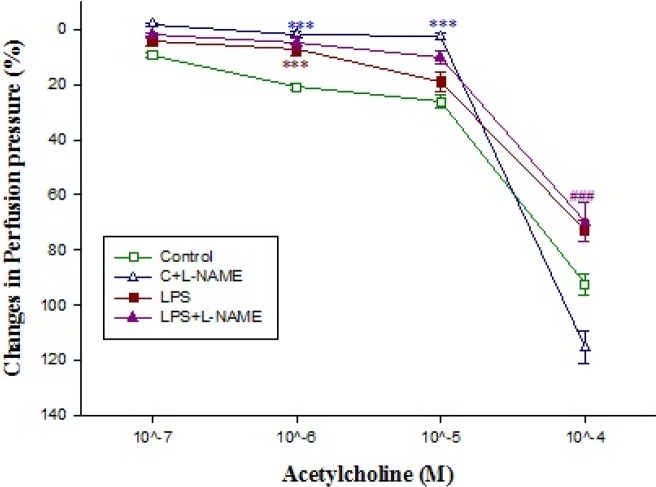
The concentration-response curves of acetylcholine -induced (10^-7^-10^-4^ M) relaxation in isolated kidney from control (C; n=10) and LPS-treated rats (n=10) in absence (n=5) and presence (n=5) of L-NAME incubation (10 μM for 20 min). Data are presented as mean±SEM. *** *P*<0.001 vs. control, ###; *P*<0.001 vs. control+L-NAME

**Figure 7:**
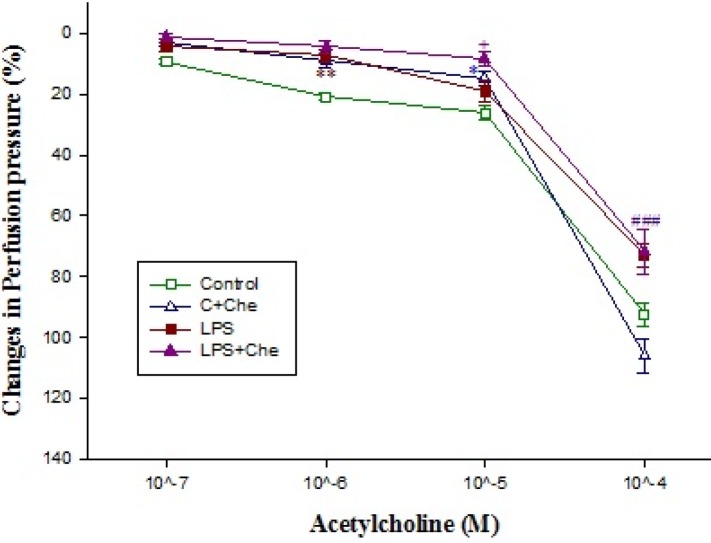
The concentration-response curves of acetylcholine -induced (10^-7^-10^-4^ M) relaxation in isolated kidney from control (C; n=10) and LPS-treated rats (n=10) in absence (n=5) and presence (n=5) of chelerythine (Che) incubation (10 μM for 20 min). Data are presented as mean±SEM. *** *P*<0.001 vs. control, ###; *P*<0.001 vs. control+ chelerythine

**Figure 8:**
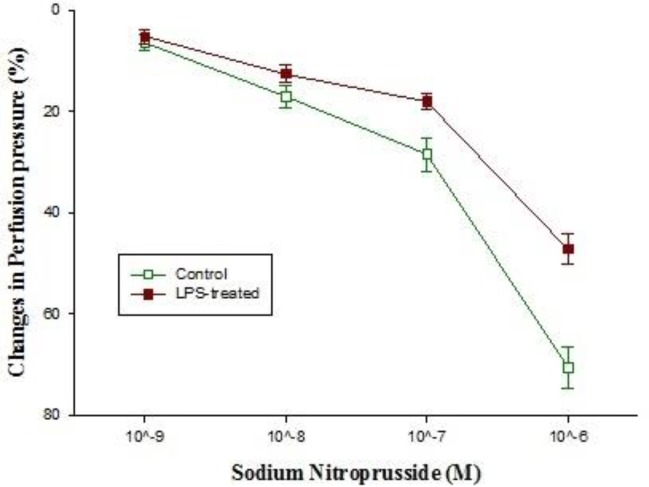
The concentration-response curves of sodium nitroprusside -induced (SNP; 10^-9^-10^-6^ M) relaxation in isolated kidney from control (n=5) and LPS-treated rats (n=5)

Our result also showed that LPS administration decreases the pressor response of isolated kidney to incremental concentrations of phenylephrine, which L-NAME incubation did not change it. Therefore, these findings may reject the hypothesis of the exclusive role of kidney’s NO overproduction in this hyporesponsiveness. It has been shown that during sepsis the amount of iNOS expression in kidney was lesser than other organs (18, 19). This indeed seems to be the case in renal responses to the vasodilators, regardless of their mechanism of action in the present septic shock model. The important role of endothelin in septic shock has also been shown (20). However, since we did not measure the amount of endothelin in this study, it would not be accurate to mention the role of endothelin in cardiovascular responses with a high degree of confidence.

We had already shown that pretreatment of either aortic rings and isolated mesenteric beds removed from DOCA-hypertensive rats with chelerythrine can significantly improve the endothelial dysfunction (21), to investigate the effectiveness of chelerythrine in other states of endothelial dysfunction, such as septic shock, isolated kidneys were preincubated with chelerythrine, a PKC inhibitor. Chelerythrine incubation improved the hyporesponsiveness to phenylephrine in LPS-treated animals which may show the probable effect of PKC in this process. Inflammatory mediators such as IL1β, TNFα, and LPS may increase a tissue factor in mesangial cells**,** which stimulate vascular thrombosis and fibrin deposition in the glomerulus. This process may be mediated by a PKC dependent pathway (22). However, chelerythrine was unable to modify the renal vascular responses to either acetylcholine or sodium nitroprusside. 

## Conclusion

The present study highlighted that five hours of intraperitoneal endotoxin injection is adequate to reduce renal basal perfusion pressure. Although our study partly elaborated on the effects of LPS on isolated renal vascular responses to vasoactive agents, further studies are required to explain how LPS exerts its renal vascular effects. 
